# The zebrafish (*Danio rerio*) anxiety test battery: comparison of behavioral responses in the novel tank diving and light–dark tasks following exposure to anxiogenic and anxiolytic compounds

**DOI:** 10.1007/s00213-021-05990-w

**Published:** 2021-10-15

**Authors:** Barbara D. Fontana, Nancy Alnassar, Matthew O. Parker

**Affiliations:** Brain and Behaviour Laboratory, School of Pharmacy and Biomedical Sciences, University of Portsmouth, Old St Michael’s Building, White Swan Road, Portsmouth, PO1 2DT UK

**Keywords:** Behavioral test battery, Conspecific alarm substance, Zebrafish, Fluoxetine

## Abstract

**Rationale:**

Triangulation of approaches (i.e., using several tests of the same construct) can be extremely useful for increasing the robustness of the findings being widely used when working with behavioral testing, especially when using rodents as a translational model. Although zebrafish are widely used in neuropharmacology research due to their high-throughput screening potential for new therapeutic drugs, behavioral test battery effects following pharmacological manipulations are still unknown.

**Methods:**

Here, we tested the effects of an anxiety test battery and test time following pharmacological manipulations in zebrafish by using two behavioral tasks: the novel tank diving task (NTT) and the light–dark test (LDT). Fluoxetine and conspecific alarm substance (CAS) were chosen to induce anxiolytic and anxiogenic-like behavior, respectively.

**Results:**

For non-drug-treated animals, no differences were observed for testing order (NTT → LDT or LDT → NTT) and there was a strong correlation between performances on the two behavioral tasks. However, we found that during drug treatment, NTT/LDT responses are affected by the tested order depending on the test time being fluoxetine effects higher at the second behavioral task (6 min later) and CAS effects lower across time.

**Conclusions:**

Overall, our data supports the use of baseline behavior assessment using this anxiety test battery. However, when working with drug exposure, data analysis must carefully consider time-drug-response and data variability across behavioral tasks.

## Introduction

In behavioral research, triangulation of approaches (i.e., using several tests of the same construct) can be extremely useful for increasing the robustness of the findings, and thus increase the confidence in the validity of the results (Stegenga 2009). In light of this, behavioral test batteries are widely employed, where animals are tested in multiple behavioral tasks either on the same day or across weeks, and the results are triangulated to gain a more robust operational definition of the target behavior (Paylor et al. 2006). While behavioral test batteries are common in rodent research, they have been less widely employed in zebrafish behavioral studies. Instead, there is a tendency to use a larger sample of animals. A drawback of this approach in zebrafish is that it increases the number of potentially unnecessary animals used in research (Born et al. 2017; McIlwain et al. 2001; Tammimäki et al. 2010). However, the systematic assessment of the impact of multiple behavioral tests on zebrafish performance in the assays is yet to be carried out.

Anxiety is a transdiagnostic trait observed across many affective disorders, and understanding more about its underlying biology would assist in the development of novel or repositioned pharmacotherapeutics (Demetriou et al. 2021; Newby et al. 2015). To address this, zebrafish (*Danio rerio)* have been widely used in the translational neuroscience of affective disorders using anxiety as a core subject of investigation (Cachat et al. 2010; Maximino et al. 2010b; Stewart et al. 2012). The two most commonly employed assays for studying anxiety-like behavior in zebrafish are the novel tank diving task (NTT) and the light–dark test (LDT) (Blaser and Rosemberg 2012). The NTT and LDT have been extensively validated using drugs that induce anxiolytic and anxiogenic effects across species, including humans (Egan et al. 2009; Parker et al. 2012; Rosemberg et al. 2012).

The NTT exploits the natural tendency of zebrafish to dive to the bottom of a novel environment, gradually exploring the top zone of the tank as they habituate to the environment (Levin et al. 2007). In the NTT, anxiety can be operationally defined in terms of (either) time spent in the bottom (↑ time =  = ↑ anxiety), in the top half (↑ time =  = ↓ anxiety), or top third (↑ time =  = ↓ anxiety) (Egan et al. 2009; Gerlai et al. 2000; Parker et al. 2012; Rosemberg et al. 2012) of a novel tank. Similarly, the LDT evaluates the extent of a fishes natural tendency for scototaxis (aversion to bright areas and natural preference for the dark) in a novel environment (Blaser and Penalosa 2011; Facciol et al. 2017). In the LDT, anxiety is operationally defined either by time spent in the light portion (↑ time =  = ↓ anxiety) or more time spent in the dark compartment (↑ time =  = ↑ anxiety) (Blaser and Rosemberg 2012; Facciol et al. 2019; Gerlai et al. 2000; Maximino et al. 2010a; Mezzomo et al. 2016). In both tasks, factors such as lighting, handling, pre-test housing, and the color of the tank play an important role in fish behavioral response (Blaser and Rosemberg 2012; Parker et al. 2012).

Song et al. (2016) evaluated the impact of carrying out the NTT and LDT as a test battery, and found no impact on baseline performance of their animals. In addition, they tested the impact of repeated testing following a mild (bright light) and strong (transportation in a car) stressor. They found no significant differences between the responses of the fish across the two test times, confirming (a) that fish showed very little evidence of a test battery effect, and (b) that this was stable even after a stress challenge. Despite these promising findings, what was not clear from the Song et al.’s (2016) study was whether the test battery effect would be observed following pharmacological manipulations: this is critical to know, as zebrafish are commonly used for psychopharmacology experiments (Cassar et al. 2020; MacRae and Peterson 2015). In addition, and critically, previous studies did not examine individual fish performance across these two behavioral tasks. Because of this, it is not clear if the group effects were also observed in individual animals, or indeed, if individual animals show robust test–retest reliability across the battery.

Here, we had three aims. First, we aimed to evaluate whether testing the same individuals on different anxiety-related tasks (NTT and LDT) would affect either their baseline behavior on the task or the effect size of two well-characterized anxiolytic and anxiogenic interventions (fluoxetine, and conspecific alarm substance; CAS) on their performance. Second, we aimed to examine correlations between NTT and LDT performance endpoints to better understand the value of test batteries for anxiety in zebrafish. Third, we looked at the effects of each drug after a time delay to differentiate if the effect is caused whether by the test battery or time effect.

## Material and methods

### Animals and experimental design

Zebrafish (AB wild-type) were bred in-house and reared in standard laboratory conditions on a re-circulating system (Aquaneering, USA) on a 14-/10-h light/dark cycle (lights on at 9:00 a.m.), pH 8.4, at ∼28.5 °C (± 1 °C) in groups of 10 animals per 2.8 L. Fish were fed three times/day with a mixture of live brine shrimp and flake food. All behavioral tests were performed between 10:00 and 15:00 h (Mon-Sun).

**Fig. 1:**
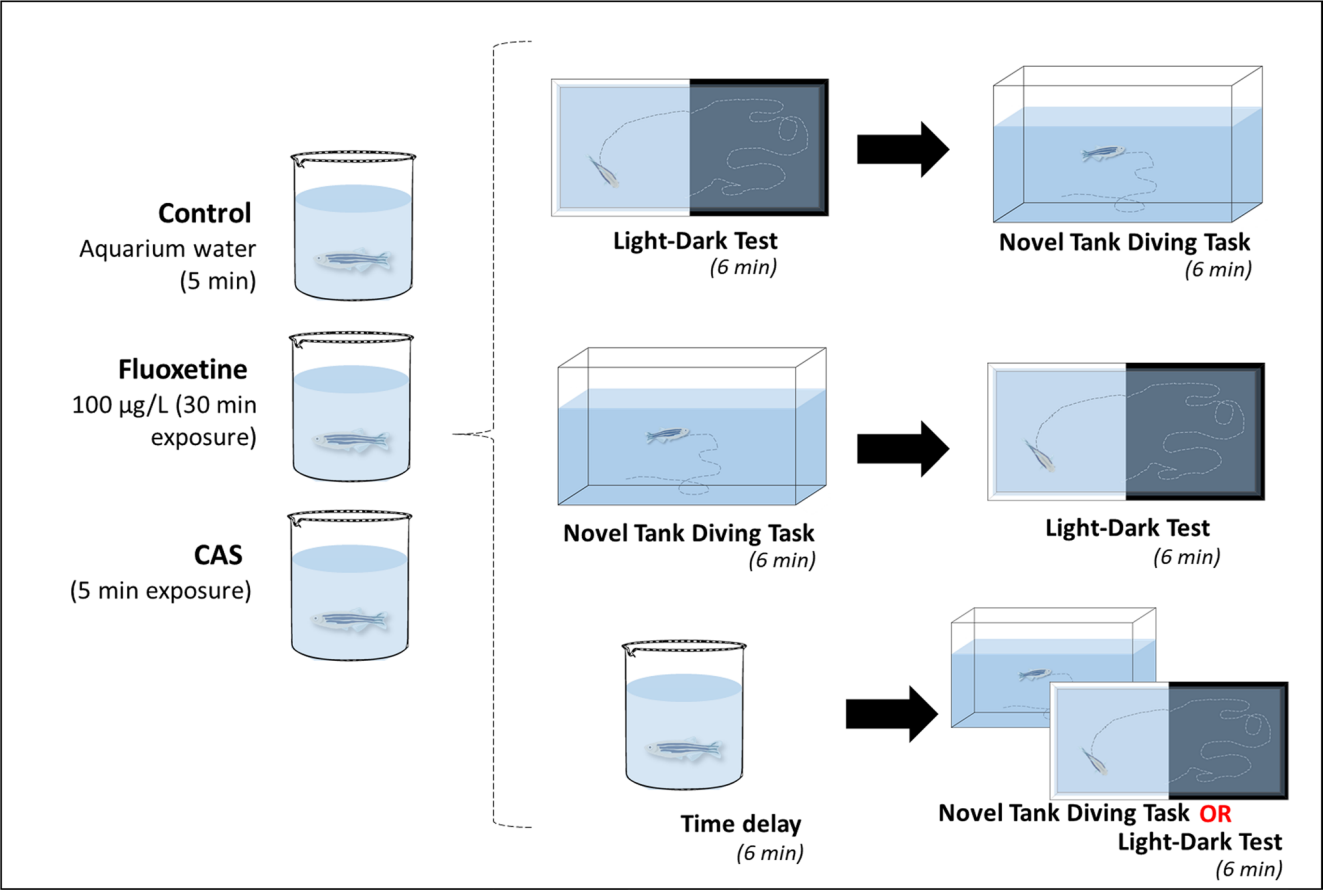
Experimental design illustration showing the behavioral test battery of NTT followed by LDT or vice-versa, and the time delay groups (6 min). For the fluoxetine and conspecific alarm substance (CAS) groups, animals were pretreated a priori behavioral assessment for 30 and 5 min, respectively

Figure 1 depicts the experimental design. Adult zebrafish (4 mpf; 50:50 female:male ratio) were first transferred to 300-mL beakers containing either aquarium-treated water for 5 min (handling control), fluoxetine (100 µg/L; 30-min exposure), or conspecific alarm substance (CAS; 5-min exposure), and then transferred either to NTT, LDT, or to new beaker (6 min—time delay groups) (see below). Animals were then immediately transferred to the second anxiety test (NTT or LDT depending on the first task assessed). Animals from the time delay group were tested only in one behavioral task. Fluoxetine was obtained from Sigma-Aldrich (Dorset, UK). After behavioral recording, fish were euthanized using 2-phenoxyethanol from Aqua-Sed (Aqua-Sed™, Vetark, Winchester, UK).

Required sample size of ~ 64 for each drug exposure (*n* = 16 NTT → LDT + *n* = 16 LDT → NTT + *n* = 16 time delayed + NTT + *n* = 16 time delayed + LDT) was calculated a priori following pilot experiments and previous sample size used for testing drug effects in the NTT and LDT in our lab (*d* = 1.25, power = 0.8, alpha = 0.05). To ensure data reliability, two independent batches were tested (choosing *n* = 8 fish from several housing tanks each batch). All behavioral testing was carried out in a fully randomized order, randomly choosing fish from one of four housing tanks for drug exposure followed by behavioral testing. After each behavioral trial, the water from the NTT, LDT, and beakers was changed. All experiments were carried out following approval from the University of Portsmouth Animal Welfare and Ethical Review Board, and under license from the UK Home Office (Animals (Scientific Procedures) Act, 1986) [PPL: P9D87106F].

### Conspecific alarm substance (CAS) extraction

CAS is a fear cue that has been successfully used to trigger stress-related responses at physiological and behavioral levels in different fish species (Abreu et al. 2016; Canzian et al. 2017; Fraker et al. 2009; Hall and Suboski 1995; Quadros et al. 2016; Speedie and Gerlai 2008; Wong et al. 2010). Briefly, CAS exposure was performed by individually exposing fish to 1.05 mL of CAS preparation in 300-mL beakers for 5 min. In order to obtain CAS, a phenotypically similar donor fish was killed using rapid cooling (submersion in 2 °C water). The epidermal cells were then cut with 10 shallow slices on both sides of the body using a razor blade. Ten milliliters of distilled water was then added into a Petri dish and mixed to fully cover the fish’s body. All procedures were performed on ice and controlled to avoid drawing blood and any external contamination (Canzian et al. 2017; Egan et al. 2009; Quadros et al. 2016; Speedie and Gerlai 2008). After CAS exposure, fish were tested in the NTT → LDT or LDT → NTT (counterbalanced 50:50).

### Novel tank diving test (NTT)

Animals (*n* = 144) were placed individually in a purpose-built transparent tank (20 cm length × 17.5 cm height × 5 cm width) containing 1 L of aquarium water. Behavioral activity was analyzed using the Zantiks AD system’s purpose-built NTT (Zantiks Ltd., Cambridge, UK) for 6 min (Egan et al. 2009; Parker et al. 2012; Rosemberg et al. 2012). The Zantiks AD system was fully controlled via a web-enabled device during behavioral training. The tank was separated into three virtual zones (bottom, middle, and top) to provide a detailed evaluation of vertical activity. The following endpoints were analyzed: distance traveled, and time spent in the top zone.

### Light–dark test (LDT)

The LDT was performed in a black tank (20 cm length × 15 cm height × 15 cm width) divided into two equally sized partitions where half of the tank area contained a bright white light and the other half was covered with a purpose-built black partition to avoid light exposure. Animals (*n* = 144) were place individually into the behavioral apparatus and their activity was analyzed using the Zantiks AD system’s purpose-built LDT equipment (Zantiks Ltd., Cambridge, UK) for 6 min to determine the time spent in the dark area (Blaser and Rosemberg 2012; Maximino et al. 2010a; Mezzomo et al. 2016).

### Statistics

Normality and homogeneity of variances were ascertained by the Kolmogorov–Smirnov and Bartlett’s test, respectively. Control groups NTT and LDT data were analyzed using one-way ANOVA (baseline behavior NTT/LDT for 1^st^ vs. 2^nd^ vs. delay (6 min)). Two-way ANOVA with multiple testing (two levels: 1^st^ vs*.* 2^nd^ tested in a new environment or 1^st^ tested vs. time delay for behavioral testing) and substance exposure (between-subjects factor—three levels: control, fluoxetine, CAS) as fixed factors were used to compare anxiety endpoints (NTT, time spent in top of the tank and distance traveled; LDT, time in the dark area). Results were expressed as means ± standard error of the mean (S.E.M). Tukey’s test was used as post-hoc analysis and all the groups were compared between each other. Results were considered significant when *p* ≤ 0.05. Heat maps were used to summarize differences between groups in the NTT or LDT comparing 1^st^, 2^nd^, and time delayed groups. Pearson correlation analysis was used to assess the association between time spent in the top zone and in the lit area for the control, fluoxetine, and CAS groups, independently of testing order.

## Results

### Multiple testing does not affect anxiety-like behavior in drug-free animals

**Fig. 2:**
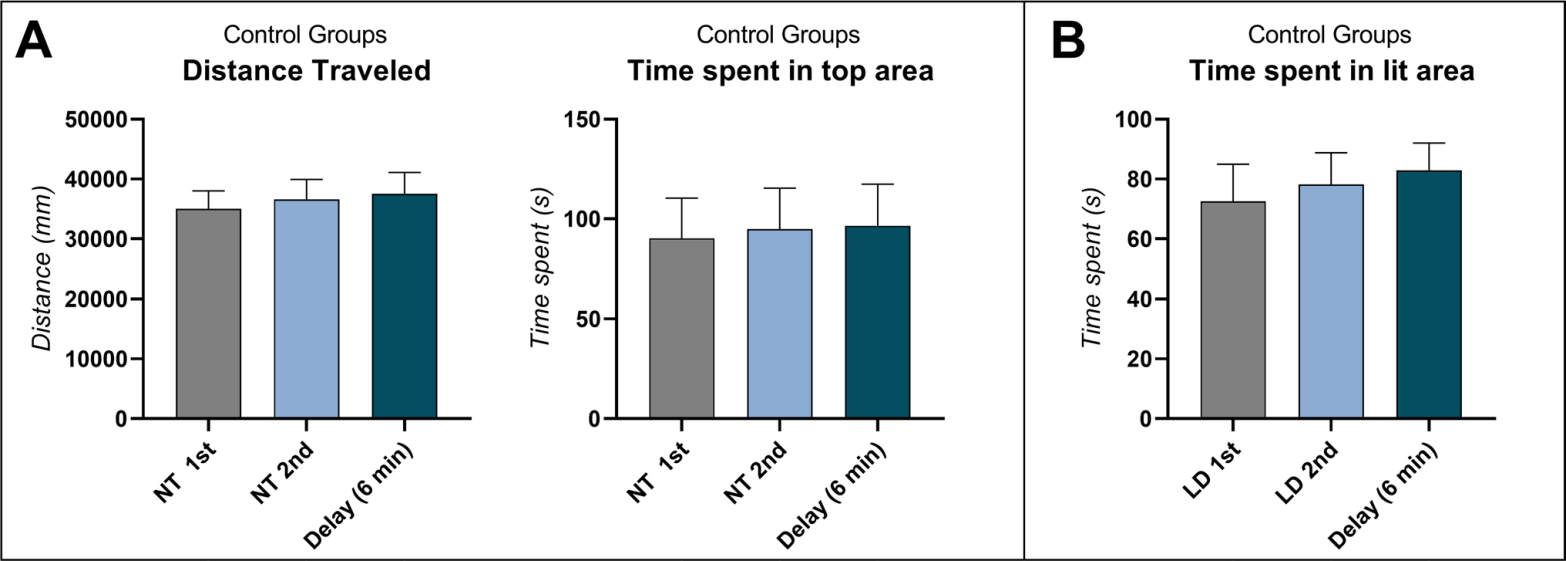
The effects of behavioral test battery and time delay in **A** novel tank diving task and **B** light–dark test of wild-type (WT) zebrafish. Data were represented as mean ± S.E.M and analyzed by *T* test (*n* = *16 per group*)

Figure 2 shows the distance traveled, time spent in top, and time spent in the lit area for control zebrafish tested in both NTT and LDT. For locomotion, no significant effect was observed for the distance traveled in the NTT (*F*_(2, 45)_ = 0.1485; *p* = 0.8624). Similarly, no significant difference was observed for controls’ time spent in top (tested 1^st^ vs. 2^nd^ vs. delay _(6 min)_; *F*_(2, 45)_ = 0.02521; *p* = 0.9751) (Fig. 2A). Regarding animals’ scototaxis, no significant difference was observed for animals tested in the light–dark test 1^st^ vs. 2^nd^ vs. delay _(6 min)_ (*F*_(2, 45)_ = 0.2282; *p* = 0.7969) (Fig. 2B).

## Fluoxetine anxiolytic-like effect is increased with a time delay or when secondly tested

**Fig. 3:**
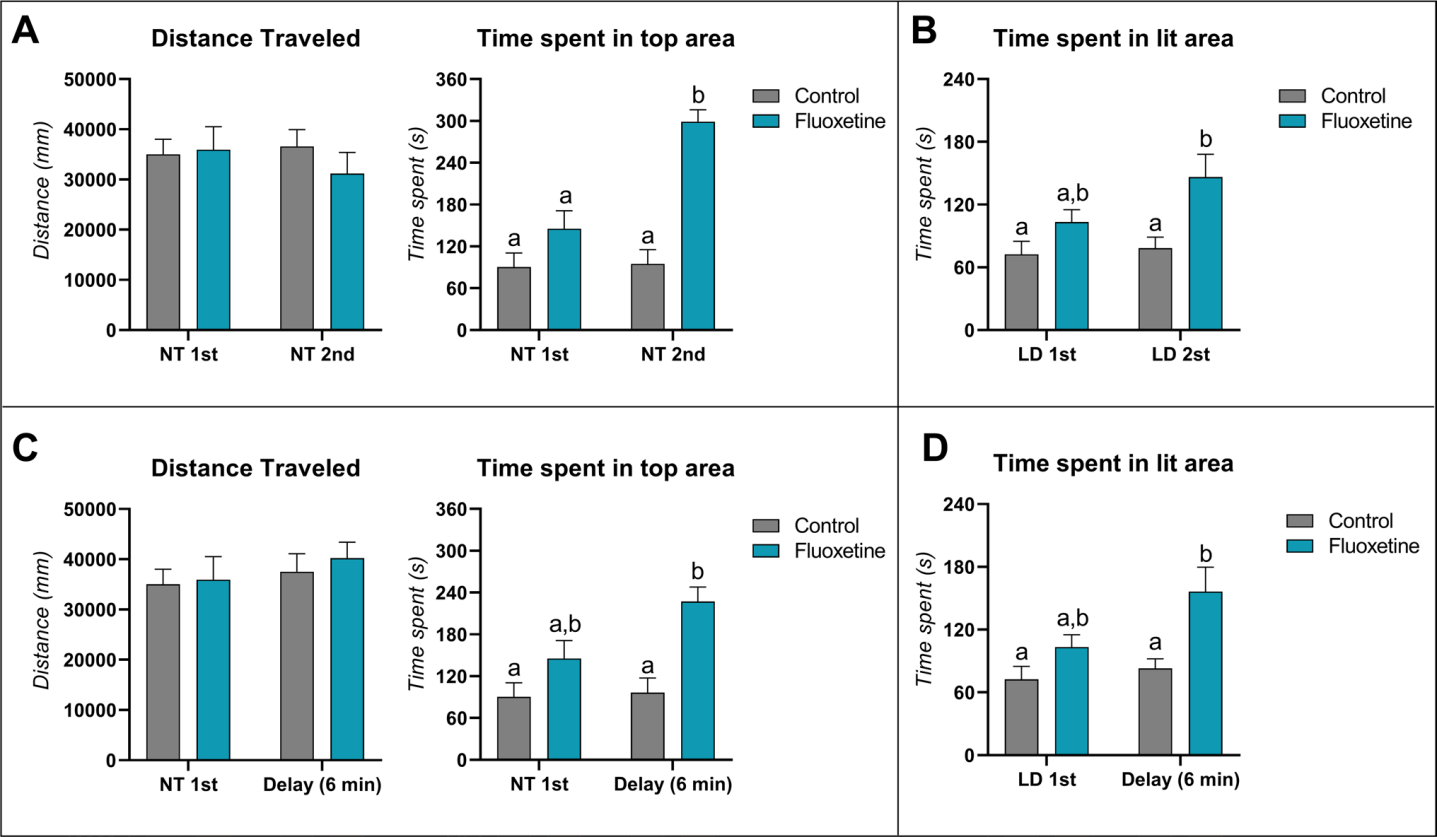
The effects of behavioral test battery in novel tank diving task (**A**) and light–dark test (**B**) of wild-type (WT) zebrafish acutely exposed to fluoxetine 100 µg/L. The effects of time delay in novel tank diving task (**C**) and light–dark test (**D**) of WT zebrafish acutely exposed to fluoxetine 100 µg/L. Data were represented as mean ± S.E.M and analyzed by two-way ANOVA (test order and fluoxetine as factors), followed by Tukey’s test multiple comparison test (*n* = *16 per group*)

In Fig. 3, the behavioral phenotype of fish exposed to fluoxetine compared to controls tested in the NTT and LDT is compared. There was no significant effect on the distance traveled in terms of test order (*F*_(1,60)_ = 0.1671; *p* = 0.6842), fluoxetine (*F*_(1,60)_ = 0.3467; *p* = 0.558), or their interaction (*F*_(1,60)_ = 0.6865; *p* = 0.4106). There was, however, a significant effect of test order (*F*_(1,60)_ = 14.00; p*** = 0.0004), fluoxetine (*F*_(1,60)_ = 37.55; p**** < 0.0001), and interaction between factors (*F*_(1,60)_ = 12.41; p*** = 0.0008) for time spent in the top zone. A significant increase in the time spent in top for fluoxetine 2^nd^ group compared to controls 1^st^ (p**** < 0.0001), fluoxetine 1^st^ (p**** < 0.0001), and controls 2^nd^ (p**** < 0.0001) was observed (Fig. 3A). Regarding the effects of fluoxetine on multiple testing in the LDT, there was a significant main effect of fluoxetine exposure (*F*_(1,60)_ = 11.01; p** = 0.0015) but no test order effect (*F*_(1,60)_ = 2.667; *p* = 0.1077). No interaction between factors (test order*fluoxetine) was observed for time spent in the lit area (*F*_(1,60)_ = 1.563; *p* = 0.2165). Tukey’s post-hoc test showed a significant effect only for fluoxetine 2^nd^ vs. controls 1^st^ (p** = 0.0053) and controls 2^nd^ (p* = 0.0120). No significant effect was observed for fluoxetine 1^st^ vs. fluoxetine 2^nd^ (*p* = 0.2456) (Fig. 3B).

Similarly, however, no significant effect was found for locomotion in time delay (*F*_(1,60)_ = 0.8742; *p* = 0.3535), fluoxetine exposure (*F*_(1,60)_ = 0.2465; *p* = 0.6214), and their interaction (*F*_(1,60)_ = 0.05946; *p* = 0.8082). A significant effect for time delay (*F*_(1,60)_ = 4.046; p* = 0.488) and fluoxetine (*F*_(1,60)_ = 17.92; p**** < 0.0001) was found for time spent in top zone. No significant effect for interaction between factors (time delay*fluoxetine; *F*_(1,60)_ = 2.977; *p* = 0.0896) was found for the time spent in top. The fluoxetine time delay group was the only group to significantly increase time spent in the top zone when compared to both control NTT 1^st^ (p*** = 0.0002) and NTT time delay control (p*** = 0.0005) (Fig. 3C). Meanwhile, for the time spent in the lit zone, fluoxetine (*F*_(1,60)_ = 11.77; p** = 0.0011) and time delay (*F*_(1,60)_ = 4.345; p* = 0.0011) had a significant effect with no interaction effect being observed (*F*_(1,60)_ = 1.976; *p* = 0.1650). However, the only group that had a significant effect compared to both controls (1^st^ LDT and time delay control) was the group fluoxetine time delay (p** = 0.0015 and p** = 0.0068, respectively) (Fig. 3D).

### CAS effects are decreased over time in a test battery and after time delay

**Fig. 4:**
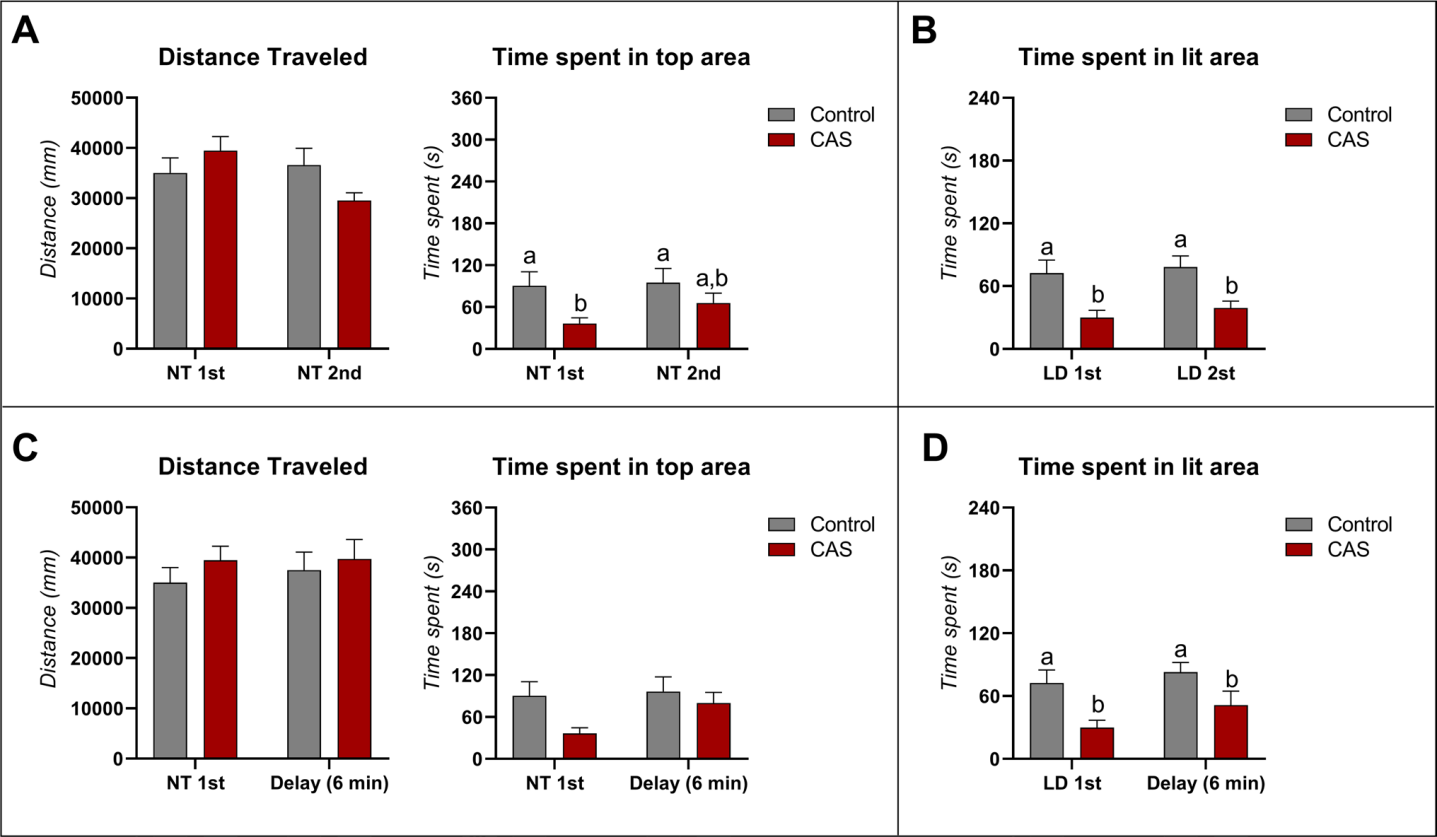
The effects of behavioral test battery in novel tank diving task (**A**) and light–dark test (**B**) of wild-type (WT) zebrafish acutely exposed to CAS for 5 min. The effects of time delay in novel tank diving task (**C**) and light–dark test (**D**) of WT zebrafish acutely exposed to CAS for 5 min. Data were represented as mean ± S.E.M and analyzed by two-way ANOVA (test order and CAS as factors), followed by Tukey’s test multiple comparison test (*n* = *16 per group*)

CAS was used as an anxiogenic control, and its effects on anxiety-like behavior in the NTT test followed by light–dark test and vice-versa are depicted in Fig. 4. A significant interaction effect (test order * CAS exposure) was observed for the distance traveled (*F*_(1,60)_ = 4.434; p* = 0.0394) but there was no main effect of test order (*F*_(1,60)_ = 2.312; *p* = 0.1336) or CAS exposure (*F*_(1,60)_ = 0.2362; *p* = 0.6287). However, no significant effect was observed through Tukey’s post-hoc analysis. Regarding animals’ time spent in the top zone, there was a significant main effect of CAS exposure (*F*_(1,60)_ = 6.326; p* = 0.0146) independent of test order. No significant interaction between factors (*F*_(1,60)_ = 0.5602; *p* = 0.4571) or test order (*F*_(1,60)_ = 1.059; *p* = 0.3077) effect was observed for CAS exposure. A significant decrease in the time spent in top was only observed for controls 1^st^ vs. CAS 1^st^ after post-hoc analysis (p* = 0.0484) and no significant difference was observed for controls 2^nd^ vs. CAS 2^nd^ (*p* = 0.3861) (Fig. 4A). Finally, although no interaction (test order vs. CAS exposure; *F*_(1,60)_ = 0.03571; *p* = 0.8508) and test order effect (*F*_(1,60)_ = 0.6211; *p* = 0.4338) were observed for time spent in the lit zone, a significant CAS exposure effect was observed (*F*_(1,60)_ = 18.47; *p* < 0.0001). Briefly, CAS exposure decreased the time spent in the lit area comparing CAS-exposed tested 1^st^ and 2^nd^ with their own controls (p* = 0.0124 and p* = 0.0257, respectively).

**Fig. 5:**
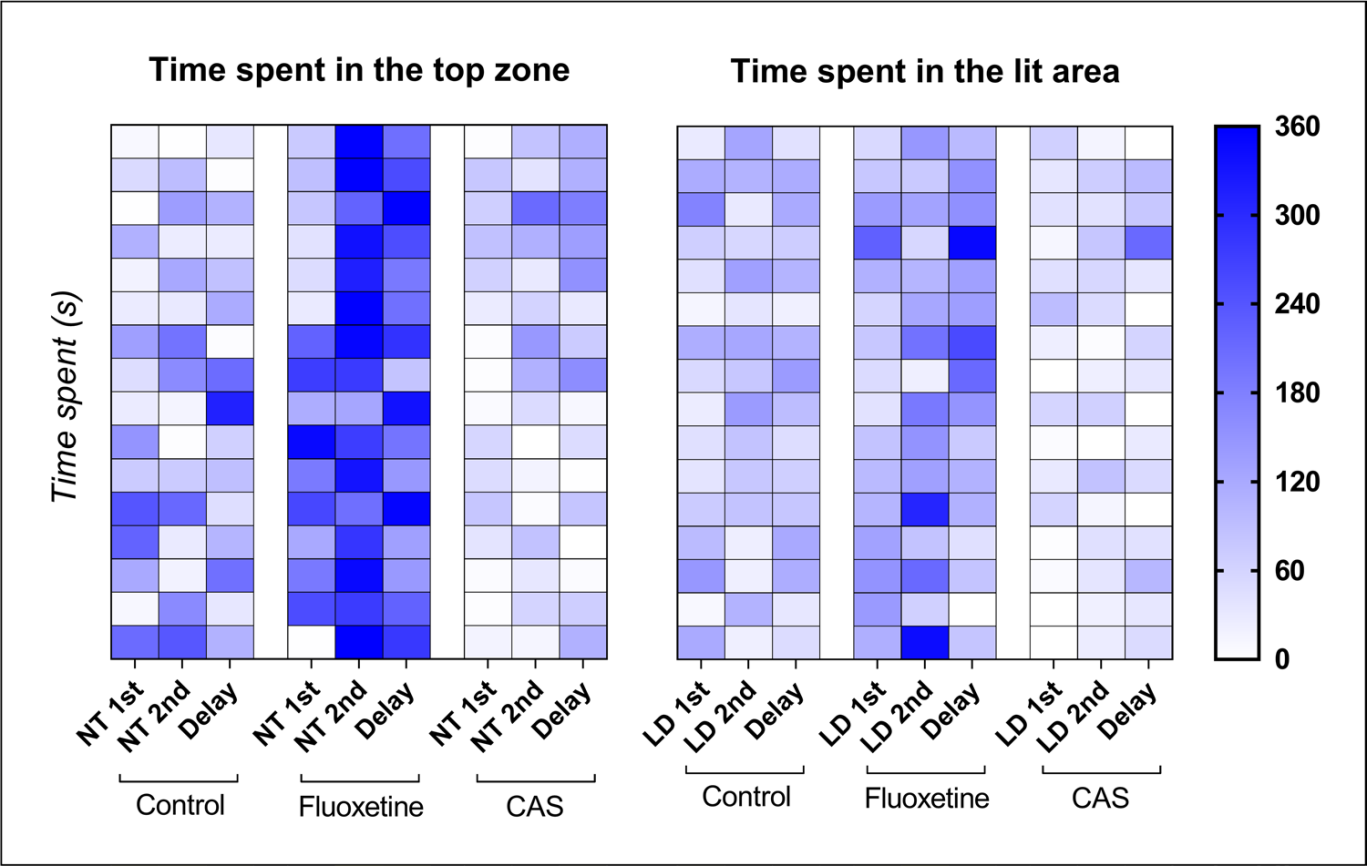
Representative heat maps showing the time spent in the top zone and in the lit area for each individual fish from different treatments (control vs. fluoxetine vs. CAS)

When looking at the effect of time delay on CAS exposure (Fig. 4C and D), a significant effect was found for CAS only when looking at the time spent in the lit zone (*F*_(1,60)_ = 11.77; p** = 0.0011) and time spent in the top zone (*F *_(1,60)_ = 4.351; p* = 0.0413). No significant effects were observed for the interaction between factors (*F*_(1,60)_ = 0.2666; *p* = 0.6075) and for time delay (*F*_(1,60)_ = 2.162; *p* = 0.1467) for the time spent in the lit zone. Meanwhile, no interaction between factors (*F*_(1,60)_ = 1.255; *p* = 0.2727) and time delay effect (*F*_(1,60)_ = 2.189; *p* = 0.1443) was observed for time spent in the top zone. Distance traveled was not affected by CAS, time delay, or interaction between factors distance traveled (*F*_(1,60)_ = 0.9922; *p* = 0.3232, *F*_(1,60)_ = 0.1734; *p* = 0.6786 and F _(1,60)_ = 0.1123; *p* = 0.7387, respectively). Tukey’s post-hoc test yielded a significant difference only for time spent in the lit zone of 1^st^ LDT + CAS group compared to both 1^st^ LDT (p* = 0.0345) and time delay LDT group (p** = 0.0053). The individual values for the time spent in the top zone and in the lit area for both drugs are summarized in Fig. 5.

### Time spent in the top and lit zones is positively correlated

**Fig. 6:**
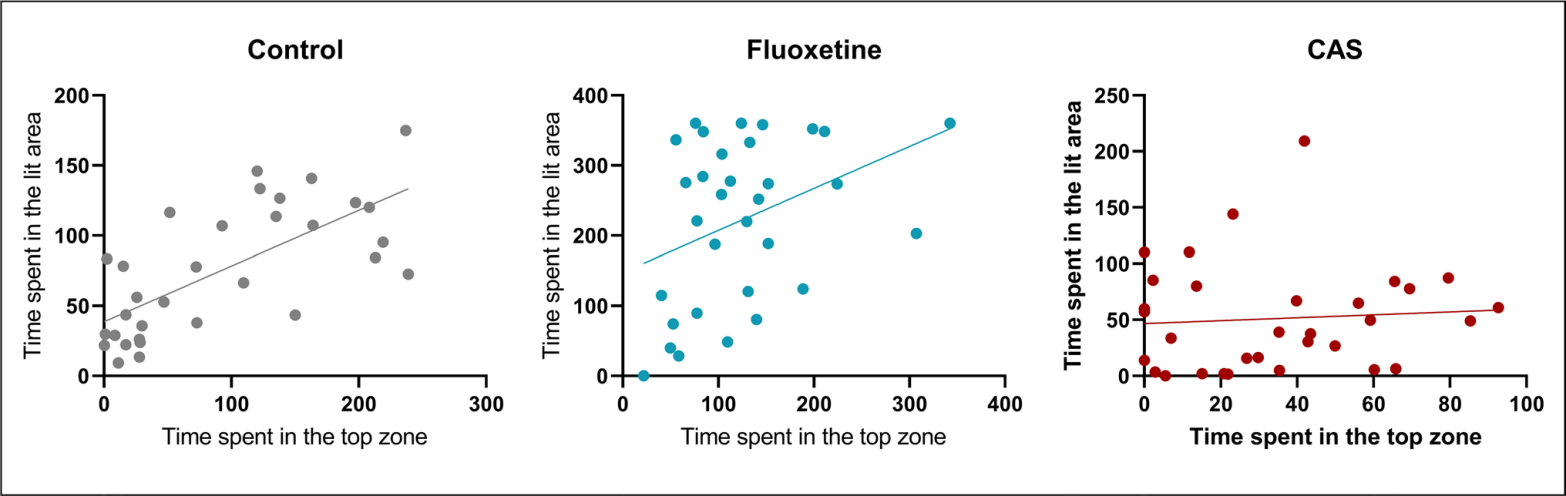
Correlational analysis between time spent in the lit area and time spent in the top zone for each group (*n* = 32)

Figure 6 displays intercorrelations between the endpoints for both the behavioral measures, both in the presence of CAS and fluoxetine, and with no-drug treatment. There was a strong positive correlation for the time spent in top (NTT) and time spent in the lit zone (LDT) for the no-drug-treated group (*r* = 0.6954; p**** < 0.0001; *n* = 32), and a moderate positive correlation for the fluoxetine group (*r* = 0.3736; p* = 0.0352; *n* = 32). However, there was no correlation between endpoints in the tests in the CAS-exposed animals (*r* = 0.0754; *p* = 0.6917; *n* = 32).

## Discussion

Here, for the first time, we tested how using the same individuals in two anxiety-related tasks affects their behavioral responses to the protocols, both in the absence of drugs and following exposure to an anxiolytic and anxiogenic compound (fluoxetine and CAS, respectively). Additionally, we examined whether introducing a time delay plays a role in the drug response of fluoxetine and CAS, or it is the test battery that increases or decreases animals’ behavioral response in a second task. We also examined, for the first time, how individuals performed across the different tasks to better understand individual performance characteristics in the two tests. We found that parameters linked to anxiety-like behavior, such as the time spent in top and time spent in the lit area, are not affected by testing wild-type (WT) fish in the NTT followed by LDT and vice-versa. However, when animals are exposed to fluoxetine, a larger effect size was observed in the second test, independent of the test (i.e*.*, NTT or LDT) which suggests that there is an impact of the time in which these fish were tested, rather than multiple testing. This hypothesis was later confirmed by testing animals in the NTT or LDT after a time delay with no previous behavioral testing. Differently from fluoxetine, CAS had its higher effect in the when immediately tested; however, similar to what was found for fluoxetine, the results were consistent, independent of the task. Moreover, lower correlation values were found when comparing the time spent in the top zone vs. time spent in the lit area for both fluoxetine- and CAS-exposed animals, suggesting higher data variability when animals are tested in both behavioral tasks and these parameters are compared independently of testing order.

The NTT and the LDT are widely used to assess anxiety-like behavior in zebrafish (Cachat et al. 2010; Maximino et al. 2010b; Stewart et al. 2012). The comparison across tasks has been previously studied, with both tasks demonstrating good cross-test correlation in vivo and similar sensitivity to zebrafish anxiety-like states (Kysil et al. 2017). In rodents, behavioral test battery is commonly used to study several behaviors including anxiety-related tasks using open field and light–dark transitions (Okuda et al. 2018). In zebrafish, studies have used a combination of social behavior, memory, and anxiety tests in order to examine inter-domain correlations, but also to minimize the use of animals (Fontana et al. 2020, 2021). Zebrafish have previously been shown to display a similar behavioral phenotype when tested in the NTT → LDT or LDT → NTT (Song et al. 2016). In the same study, an acute stressor (30-min car transportation) increased anxiety-related patterns in both tasks (Song et al. 2016). These data suggest that a strong effect can still be observed when performing behavioral test batteries in zebrafish, with no impact of test order or multiple testing by itself. Here, we found that the overall baseline response of our control WT zebrafish is kept the same across tasks when testing NTT and LDT, which supports the use of this species across anxiety-related tasks in order to reduce the number of animals used in research. However, when animals were acutely exposed to CAS or fluoxetine, different effects were observed when performing a behavioral test battery.

A significant role for fluoxetine in decreasing anxiety is more pronounced in the 2^nd^ behavioral task or after 6 min of time delay, independently of it being NTT or LDT. No significant differences were observed in the 1^st^ behavioral task. Fluoxetine is a selective serotonin reuptake inhibitor (SSRI) commonly used to treat several psychiatric disorders in humans. The role of fluoxetine in zebrafish anxiety is somewhat controversial. For example, Stewart et al. (2011b) showed that fluoxetine had no effect on anxiety-like behavior at a concentration of 0.1 mg/L (the same concentration used here). However, these animals had an increased tendency to spend time in the top zone of the tank, which can be an indicator of decreased anxiety. The authors discussed that the lack of anxiolytic effects following acute fluoxetine in zebrafish contradicts clinical and rodent findings (Hascoët et al. 2000; Lightowler et al. 1994; Varty et al. 2002). Here, the anxiolytic effect of fluoxetine was only observed when fish were tested in the second task or after 6 min. This could explain the data variability across papers, since we observed no effect when fish were immediately tested in the NTT or LDT. Altogether, these data suggest that there is a temporal delay in the effects of fluoxetine. The pharmacokinetics and pharmacodynamics of fluoxetine vary depending on administration route and time/duration of its exposure (Caccia et al. 1990; Sawyer and Howell 2011). For example, a study with non-human primates showed that the peak of fluoxetine in serum is achieved at different times depending on the drug concentration (15 min for 31 ng/mL, 30 min for 70 ng/mL, and 60 min for 165 ng/mL). Meanwhile, its main metabolite, norfluoxetine, was only found at 120 min for all the doses tested lasting up to 24 h after fluoxetine exposure (Sawyer and Howell 2011). Although fluoxetine has been commonly used as an anxiolytic drug in neuropsychiatric studies using zebrafish, time-dose–response studies looking at the effects of fluoxetine using water exposure as the administration route in this species is lacking. A recent study has explored the long-term effects of fluoxetine in zebrafish behavior up to 28 days after acute exposure to different concentrations, where the authors found that the fluoxetine effects vary depending on time of behavioral testing (Al Shuraiqi et al. 2021). However, fluoxetine short time-dose–response in anxiety-related paradigms is still unknown being an important step for the understanding of data variability across labs and the mechanisms underlying fluoxetine inducing anxiolytic and anxiogenic phenotypes.

Although a significant decrease in the time spent in the lit area could be observed when animals were tested 1^st^, 2^nd^, or after time delay in the LDT, in the NTT, no significant effects were observed between controls and CAS-exposed animals when tested 2^nd^ or after time delay. CAS is an effective acute stressor which is produced and stored in the epidermal “club” cells and is naturally released into the water after skin injuries provoked by predator bouts (Chivers and Smith 1994; Korpi and Wisenden 2001). The different concentrations and effects of CAS on zebrafish fear and anxiety-related behavior were first described by Speedie and Gerlai (2008) being its anxiogenic effects well-characterized in behavioral neuroscience research. For example, in the light–dark test, zebrafish exposed to CAS for 5 min showed increased scototaxis (preference for dark areas) (Abreu et al. 2016; Quadros et al. 2016) which is a behavioral change often observed after the exposure to anxiogenic drugs (Stewart et al. 2011a). Similarly, we found that CAS significantly decreased the time spent in the lit area and in the top zone, which indicates an increased “anxious” response. Interestingly, we found that this effect is attenuated in the second task only when NTT is the second behavioral analysis in the test battery, where a strong effect is maintained across tasks for the LDT. Similarly, when considering time delay as a factor, no significant differences were observed for CAS in the NTT, but a strong effect was still observed in the LDT even after a time delay. However, in both behavioral tasks, the effect size of CAS-induced anxiogenic behavior is decreased when animals are tested after 6 min suggesting that these effects could potentially decrease across time.

When looking at the correlation between these tasks, independent of the test order, there was a strong positive correlation between the time spent in the top zone and time spent in the lit area, making the behavioral phenotypes in those tasks comparable. Similarly, a previous study has showed that those tasks show good cross-test correlation with NTT only differing from LDT in terms of cortisol responses after tasks where NTT is correlated to higher stress-related responses (Kysil et al. 2017). In addition, here, we showed that the correlation between NTT and LDT anxiety-related variables is not always good showing low values when animals are exposed to different molecules such as fluoxetine and CAS. Although this data could indicate that data is less reliable when comparing the animals’ response in both tasks (NTT → LDT or LDT → NTT), the main effects for these drugs when tested firstly (CAS) and secondly (fluoxetine) were similar across behavioral tasks.

## Conclusion

Overall, the use of behavioral battery testing for anxiety-like behavior can indeed influence behavioral response when fish are previously exposed to a chemical substance, such as CAS or fluoxetine. However, our data indicate that the effects are not caused by the test battery per se but rather by the test time. For example, fluoxetine has higher anxiolytic-like effects when tested secondly or after a time delay. Meanwhile, CAS effects are higher in the first behavior task compared to the second behavioral task or after 6 min. Importantly, WT behavior was not influenced by testing animals in both new environments. Our findings may be particularly important for characterization of mutant lines, where a reduced number of animals could potentially be used to evaluate baseline behavior when there is no influence of drug exposure. However, further studies are still necessary to compare data between WT animals and genetically altered fish. Altogether, this supports the use of baseline behavior assessment using multiple tasks; however, researchers must carefully prepare their experimental design when testing drugs and conducting behavioral battery considering the drug time-dose–response.

## References

[CR1] Abreu MS, Giacomini AC, Gusso D, Koakoski G, Oliveira TA, Marqueze A, Barreto RE, Barcellos LJ (2016). Behavioral responses of zebrafish depend on the type of threatening chemical cues. J Comp Physiol A Neuroethol Sens Neural Behav Physiol.

[CR2] Al Shuraiqi A, Al-Habsi A, Barry MJ (2021). Time-, dose- and transgenerational effects of fluoxetine on the behavioural responses of zebrafish to a conspecific alarm substance. Environ Pollut.

[CR3] Blaser RE, Penalosa YM (2011). Stimuli affecting zebrafish (Danio rerio) behavior in the light/dark preference test. Physiol Behav.

[CR4] Blaser RE, Rosemberg DB (2012). Measures of anxiety in zebrafish (Danio rerio): dissociation of black/white preference and novel tank test. PLoS One.

[CR5] Born HA, Dao AT, Levine AT, Lee WL, Mehta NM, Mehra S, Weeber EJ, Anderson AE (2017). Strain-dependence of the Angelman syndrome phenotypes in Ube3a maternal deficiency mice. Sci Rep.

[CR6] Caccia S, Cappi M, Fracasso C, Garattini S (1990). Influence of dose and route of administration on the kinetics of fluoxetine and its metabolite norfluoxetine in the rat. Psychopharmacology.

[CR7] Cachat J, Stewart A, Grossman L, Gaikwad S, Kadri F, Chung KM, Wu N, Wong K, Roy S, Suciu C, Goodspeed J, Elegante M, Bartels B, Elkhayat S, Tien D, Tan J, Denmark A, Gilder T, Kyzar E, Dileo J, Frank K, Chang K, Utterback E, Hart P, Kalueff AV (2010). Measuring behavioral and endocrine responses to novelty stress in adult zebrafish. Nat Protoc.

[CR8] Canzian J, Fontana BD, Quadros VA, Rosemberg DB (2017). Conspecific alarm substance differently alters group behavior of zebrafish populations: putative involvement of cholinergic and purinergic signaling in anxiety- and fear-like responses. Behav Brain Res.

[CR9] Cassar S, Adatto I, Freeman JL, Gamse JT, Iturria I, Lawrence C, Muriana A, Peterson RT, Van Cruchten S, Zon LI (2020). Use of zebrafish in drug discovery toxicology. Chem Res Toxicol.

[CR10] Chivers DP, Smith RJ (1994). Intra- and interspecific avoidance of areas marked with skin extract from brook sticklebacks (Culaea inconstans) in a natural habitat. J Chem Ecol.

[CR11] Demetriou EA, Park SH, Pepper KL, Naismith SL, Song YJ, Thomas EE, Hickie IB, Guastella AJ (2021). A transdiagnostic examination of anxiety and stress on executive function outcomes in disorders with social impairment. J Affect Disord.

[CR12] Egan RJ, Bergner CL, Hart PC, Cachat JM, Canavello PR, Elegante MF, Elkhayat SI, Bartels BK, Tien AK, Tien DH, Mohnot S, Beeson E, Glasgow E, Amri H, Zukowska Z, Kalueff AV (2009). Understanding behavioral and physiological phenotypes of stress and anxiety in zebrafish. Behav Brain Res.

[CR13] Facciol A, Iqbal M, Eada A, Tran S, Gerlai R (2019). The light-dark task in zebrafish confuses two distinct factors: Interaction between background shade and illumination level preference. Pharmacol Biochem Behav.

[CR14] Facciol A, Tran S, Gerlai R (2017). Re-examining the factors affecting choice in the light-dark preference test in zebrafish. Behav Brain Res.

[CR15] Fontana BD, Cleal M, Parker MO (2020). Female adult zebrafish (Danio rerio) show higher levels of anxiety-like behavior than males, but do not differ in learning and memory capacity. Eur J Neurosci.

[CR16] Fontana BD, Gibbon AJ, Cleal M, Norton WHJ, Parker MO (2021). Chronic unpredictable early-life stress (CUELS) protocol: Early-life stress changes anxiety levels of adult zebrafish. Prog Neuropsychopharmacol Biol Psychiatry.

[CR17] Fraker ME, Hu F, Cuddapah V, McCollum SA, Relyea RA, Hempel J, Denver RJ (2009). Characterization of an alarm pheromone secreted by amphibian tadpoles that induces behavioral inhibition and suppression of the neuroendocrine stress axis. Horm Behav.

[CR18] Gerlai R, Lahav M, Guo S, Rosenthal A (2000). Drinks like a fish: zebra fish (Danio rerio) as a behavior genetic model to study alcohol effects. Pharmacol Biochem Behav.

[CR19] Hall D, Suboski MD (1995). Visual and olfactory stimuli in learned release of alarm reactions by zebra danio fish (Brachydanio rerio). Neurobiol Learn Mem.

[CR20] Hascoët M, Bourin M, Colombel MC, Fiocco AJ, Baker GB (2000). Anxiolytic-like effects of antidepressants after acute administration in a four-plate test in mice. Pharmacol Biochem Behav.

[CR21] Korpi NL, Wisenden BD (2001). Learned recognition of novel predator odour by Zebra Danios, Danio Rerio, following time-shifted presentation of alarm cue and predator odour. Environ Biol Fishes.

[CR22] Kysil EV, Meshalkina DA, Frick EE, Echevarria DJ, Rosemberg DB, Maximino C, Lima MG, Abreu MS, Giacomini AC, Barcellos LJG, Song C, Kalueff AV (2017). Comparative analyses of zebrafish anxiety-like behavior using conflict-based novelty tests. Zebrafish.

[CR23] Levin ED, Bencan Z, Cerutti DT (2007) Anxiolytic effects of nicotine in zebrafish. Physiol Behav 90(1):54–58. 10.1016/j.physbeh.2006.08.026 10.1016/j.physbeh.2006.08.026 17049956

[CR24] Lightowler S, Kennett GA, Williamson IJR, Blackburn TP, Tulloch IF (1994). Anxiolytic-like effect of paroxetine in a rat social interaction test. Pharmacol Biochem Behav.

[CR25] MacRae CA, Peterson RT (2015). Zebrafish as tools for drug discovery. Nat Rev Drug Discov.

[CR26] Maximino C, de Brito TM, Colmanetti R, Pontes AA, de Castro HM, de Lacerda RI, Morato S, Gouveia A (2010). Parametric analyses of anxiety in zebrafish scototaxis. Behav Brain Res.

[CR27] Maximino C, de Brito TM, da Silva Batista AW, Herculano AM, Morato S, Gouveia A (2010). Measuring anxiety in zebrafish: a critical review. Behav Brain Res.

[CR28] McIlwain KL, Merriweather MY, Yuva-Paylor LA, Paylor R (2001). The use of behavioral test batteries: Effects of training history. Physiol Behav.

[CR29] Mezzomo NJ, Silveira A, Giuliani GS, Quadros VA, Rosemberg DB (2016). The role of taurine on anxiety-like behaviors in zebrafish: A comparative study using the novel tank and the light-dark tasks. Neurosci Lett.

[CR30] Newby JM, McKinnon A, Kuyken W, Gilbody S, Dalgleish T (2015). Systematic review and meta-analysis of transdiagnostic psychological treatments for anxiety and depressive disorders in adulthood. Clin Psychol Rev.

[CR31] Okuda K, Takao K, Watanabe A, Miyakawa T, Mizuguchi M, Tanaka T (2018). Comprehensive behavioral analysis of the Cdkl5 knockout mice revealed significant enhancement in anxiety- and fear-related behaviors and impairment in both acquisition and long-term retention of spatial reference memory. PLOS ONE.

[CR32] Parker MO, Millington ME, Combe FJ, Brennan CH (2012). Housing conditions differentially affect physiological and behavioural stress responses of zebrafish, as well as the response to anxiolytics. PLoS One.

[CR33] Paylor R, Spencer CM, Yuva-Paylor LA, Pieke-Dahl S (2006). The use of behavioral test batteries, II: Effect of test interval. Physiol Behav.

[CR34] Quadros VA, Silveira A, Giuliani GS, Didonet F, Silveira AS, Nunes ME, Silva TO, Loro VL, Rosemberg DB (2016). Strain- and context-dependent behavioural responses of acute alarm substance exposure in zebrafish. Behav Processes.

[CR35] Rosemberg DB, Braga MM, Rico EP, Loss CM, Cordova SD, Mussulini BH, Blaser RE, Leite CE, Campos MM, Dias RD, Calcagnotto ME, de Oliveira DL, Souza DO (2012). Behavioral effects of taurine pretreatment in zebrafish acutely exposed to ethanol. Neuropharmacology.

[CR36] Sawyer EK, Howell LL (2011). Pharmacokinetics of fluoxetine in rhesus macaques following multiple routes of administration. Pharmacology.

[CR37] Song C, Yang L, Wang J, Chen P, Li S, Liu Y, Nguyen M, Kaluyeva A, Kyzar EJ, Gaikwad S, Kalueff AV (2016). Building neurophenomics in zebrafish: effects of prior testing stress and test batteries. Behav Brain Res.

[CR38] Speedie N, Gerlai R (2008). Alarm substance induced behavioral responses in zebrafish (Danio rerio). Behav Brain Res.

[CR39] Stegenga J (2009). Robustness, discordance, and relevance. Philosophy of Science.

[CR40] Stewart A, Gaikwad S, Kyzar E, Green J, Roth A, Kalueff AV (2012). Modeling anxiety using adult zebrafish: a conceptual review. Neuropharmacology.

[CR41] Stewart A, Maximino C, Marques de Brito T, Herculano AM, Gouveia A, Morato S, Cachat JM, Gaikwad S, Elegante MF, Hart PC, Kalueff AV, Kalueff AV, Cachat JM (2011). Neurophenotyping of adult zebrafish using the light/dark box paradigm. Zebrafish Neurobehavioral Protocols.

[CR42] Stewart A, Wu N, Cachat J, Hart P, Gaikwad S, Wong K, Utterback E, Gilder T, Kyzar E, Newman A, Carlos D, Chang K, Hook M, Rhymes C, Caffery M, Greenberg M, Zadina J, Kalueff AV (2011). Pharmacological modulation of anxiety-like phenotypes in adult zebrafish behavioral models. Prog Neuropsychopharmacol Biol Psychiatry.

[CR43] Tammimäki A, Käenmäki M, Kambur O, Kulesskaya N, Keisala T, Karvonen E, García-Horsman JA, Rauvala H, Männistö PT (2010). Effect of S-COMT deficiency on behavior and extracellular brain dopamine concentrations in mice. Psychopharmacology.

[CR44] Varty GB, Morgan CA, Cohen-Williams ME, Coffin VL, Carey GJ (2002). The Gerbil Elevated Plus-Maze I: behavioral characterization and pharmacological validation. Neuropsychopharmacology.

[CR45] Wong K, Elegante M, Bartels B, Elkhayat S, Tien D, Roy S, Goodspeed J, Suciu C, Tan J, Grimes C, Chung A, Rosenberg M, Gaikwad S, Denmark A, Jackson A, Kadri F, Chung KM, Stewart A, Gilder T, Beeson E, Zapolsky I, Wu N, Cachat J, Kalueff AV (2010). Analyzing habituation responses to novelty in zebrafish (Danio rerio). Behav Brain Res.

